# Development of a high-throughput UHPLC-MS/MS method for the analysis of *Fusarium* and *Alternaria* toxins in cereals and cereal-based food

**DOI:** 10.1007/s00216-024-05486-4

**Published:** 2024-09-02

**Authors:** Fabian Dick, Alena Dietz, Stefan Asam, Michael Rychlik

**Affiliations:** Chair of Analytical Food Chemistry, Technical University of Munich, Maximus-Von-Imhof Forum 2, 85354 Freising, Germany

**Keywords:** *Alternaria*, *Fusarium*, Modified mycotoxins, Cereals, QuEChERS, LC–MS/MS analysis

## Abstract

A QuEChERS (quick, easy, cheap, effective, rugged, and safe)-based multi-mycotoxin method was developed, analyzing 24 (17 free and 7 modified) *Alternaria* and *Fusarium* toxins in cereals via ultrahigh-performance liquid chromatography-tandem mass spectrometry (UHPLC-MS/MS). A modified QuEChERS approach was optimized for sample preparation. Quantification was conducted using a combination of stable isotope dilution analysis (SIDA) for nine toxins and matrix-matched calibration for ten toxins. Quantification via a structurally similar internal standard was conducted for four analytes. Alternariol-9-sulfate (AOH-9-S) was measured qualitatively. Limits of detection (LODs) were between 0.004 µg/kg for enniatin A1 (ENN A1) and 3.16 µg/kg for nivalenol (NIV), while the limits of quantification were between 0.013 and 11.8 µg/kg, respectively. The method was successfully applied to analyze 136 cereals and cereal-based foods, including 28 cereal-based infant food products. The analyzed samples were frequently contaminated with *Alternaria* toxins, proving their ubiquitous occurrence. Interestingly, in many of those samples, some modified *Alternaria* toxins occurred, mainly alternariol-3-sulfate (AOH-3-S) and alternariol monomethyl ether-3-sulfate (AME-3-S), thus highlighting the importance of including modified mycotoxins in the routine analysis as they may significantly add to the total exposure of their parent toxins. Over 95% of the analyzed samples were contaminated with at least one toxin. Despite the general contamination, no maximum or indicative levels were exceeded.

**Graphical Abstract:**

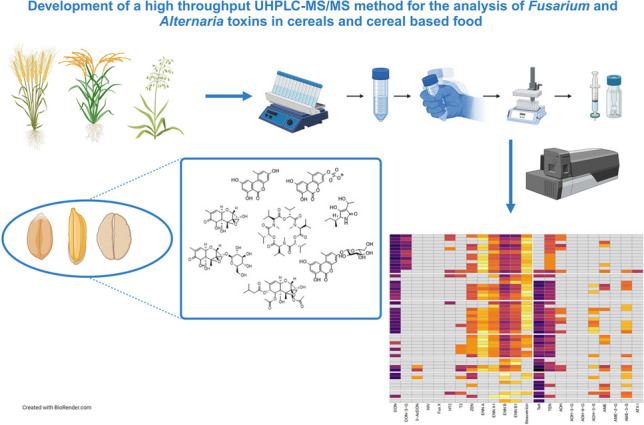

**Supplementary Information:**

The online version contains supplementary material available at 10.1007/s00216-024-05486-4.

## Introduction

According to a general definition, “mycotoxins are toxic, low molecular, secondary metabolites produced by fungi. Toxigenic fungi often grow on edible plants, contaminating food and feed” [1]. Most mycotoxin producers belong to the genera *Aspergillus*, *Alternaria*, *Fusarium*, and *Penicillium* [2]. Cereals are often infected with *Alternaria* and *Fusarium* species (sp.) and contaminated by their mycotoxins, on the analysis of which we focused in this study. While *Fusarium* toxins are routinely analyzed in cereals and resistance to *Fusarium* infection is a breeding trait, *Alternaria* toxins are not yet regulated in cereals [3, 4]. *Fusarium* sp. typically infect plants during germination, growth, and flowering stages, while *Alternaria* infection is usually favored by adverse conditions such as bad weather, pest infection, or physical damage. *Alternaria* sp. typically infect the crop after ripening or during storage [5, 6]. Therefore, simultaneous infection of a plant with *Alternaria* and *Fusarium* sp. is considered likely, and co-contamination with their toxins may be expected. The co-occurrence of mycotoxins may lead to synergistic, antagonistic, or additive effects [7], thus highlighting the importance of multi-mycotoxin methods to determine structurally different mycotoxins, often produced by different toxigenic fungi simultaneously, for an in-depth risk assessment.

**Fig. 1:**
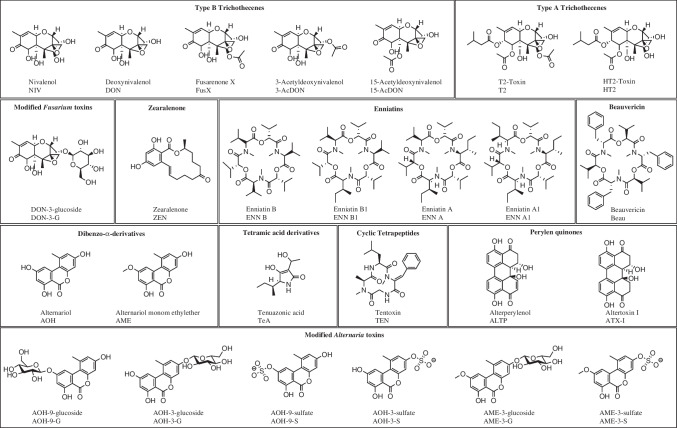
Structures of the analyzed toxins grouped by their molecular structure

Mycotoxins can be classified based on their structural characteristics, toxicity, origin, occurrence, or regulation. In the study presented here, the analyzed mycotoxins are divided into three groups: the regulated, emerging, and modified mycotoxins (for structures, see Fig. 1).

Regulated mycotoxins are the first group and have regulatory limits in food in many parts of the world. The European Commission (EC) has established maximum levels (ML) for mycotoxins by Commission Regulation (EU) 2023/915 [3]. Regulated in cereals are aflatoxins, deoxynivalenol (DON), fumonisins, ochratoxin A, zearalenone (ZEN), and the type A trichothecenes T-2 toxin (T-2) and HT-2 toxin (HT-2), for the latter of which MLs were recently introduced [8].

The second group are the emerging mycotoxins, referred to as “mycotoxins, which are neither routinely determined nor legislatively regulated” [9, 10]. Emerging *Fusarium* mycotoxins are toxins like the enniatins A, A1, B, and B1 (ENN A, A1, B, and B1), beauvericin (BEA), nivalenol (NIV), and fusarenone X (Fus X). Tentoxin (TEN) and altertoxin I (ATX I) are representative *Alternaria* toxins in this group. Indicative levels (ILs) have been introduced for other toxins, which fall between regulated and emerging mycotoxins. If these ILs are exceeded, member states of the European Union (EU) and the food business operators are required to investigate the source of the contamination. Guidance values are recommended for the *Alternaria* toxins alternariol (AOH), alternariol monomethyl ether (AME), and tenua-zonic acid (TeA) in cereal-based infant food [11].

The third group are the modified mycotoxins, of which one group is the biologically modified mycotoxins [12]. Biologically modified mycotoxins can be conjugated by microorganisms, plants, or animals. Plants usually conjugate the mycotoxins by coupling them to glucose, sulfate, or acetate, which may alter bioavailability and bioaccessibility. The modified mycotoxins may be less, equal, or even more toxic than their native toxin. Toxicological studies are mainly done on DON and ZEN conjugates, while data on other modified mycotoxins remain rare [13]. To gain more insight into the occurrence of modified mycotoxins, we implemented DON-3-glucoside (DON-3-G), AOH-3-glucoside (AOH-3-G), AOH-9-glucoside (AOH-9-G), AOH-3-sulfate (AOH-3-S), AOH-9-sulfate (AOH-9-S), AME-3-glucoside (AME-3-G), and AME-3-sulfate (AME-3-S) in the analytical method. DON-3-G is essential as it is known to significantly add to the total exposure of DON [14]. The occurrence of modified *Alternaria* toxins has not been explored well. Therefore, their inclusion in routine measurements could provide interesting insights into their occurrence. Whether the acetylated derivatives of DON (3- and 15-AcDON) are to be classified as free or modified mycotoxins is still a matter of discussion [12]. As precursors of DON in the fungal biosynthesis and not modified afterward, we categorized them as free mycotoxins.

Ultrahigh-performance liquid chromatography coupled to tandem mass spectrometry (UHPLC-MS/MS) using stable isotope dilution analysis (SIDA) is considered state-of-the-art in quantifying mycotoxins. These analytes are most commonly extracted with acetonitrile/water (ACN/H_2_O) mixtures, and post-extraction clean-up is necessary to reduce matrix effects and, if possible, to concentrate the analytes. Solid phase extraction (SPE) is often used in multi-mycotoxin analysis. While dilute-and-shoot methods may not be sensitive enough for all toxins, immunoaffinity columns (IACs) are usually very expensive and not available for all toxins. Depending on the sensitivity, matrix, and analyte requirements, SPE, IAC, dilute-and-shoot, and QuEChERS (quick, easy, cheap, effective, rugged, and safe) are often used for clean-up, with the QuEChERS approach becoming increasingly popular. Initially, it has been developed for the analysis of pesticides [15] and is applicable mainly for analyzing many small organic compounds showing a broad range of properties. However, it also allows the simultaneous separation of interfering matrix components like proteins, sugars, and lipids from the extracts. Apart from its initial applications, QuEChERS-based methods have become increasingly important for analyzing emerging and regulated mycotoxins [16].

Multi-mycotoxin methods allow keeping track of regulated mycotoxins such as DON while gaining more insight into the occurrence of emerging mycotoxins such as TeA, AOH, and AME, and modified mycotoxins such as DON-3-G, AOH-3-G, and AME-3-G. The first aim of this study, therefore, was to unify the two separate mycotoxin approaches previously applied by our group [17, 18] for the analysis of *Fusarium* and *Alternaria* toxins (Fig. 1) in one sample workup and one UHPLC-MS/MS measurement.

After method development and validation, the second aim was to apply the method to a wide range of cereal and cereal-based products, including infant foods, to gain more information on toxin occurrence and the co-occurrence of *Alternaria* and *Fusarium* toxins.

## Materials and methods

### Reagents and chemicals

Ultrapure H_2_O (both LC–MS and HPLC grade) and ACN (HPLC grade) were purchased from Th. Geyer (Renningen, Germany). Methanol (MeOH) (LC–MS grade) and ACN (LC–MS grade) were purchased from Honeywell Riedel–de Haen (Seelze, Germany). Sodium chloride (NaCl) and ammonia solution (NH_4_OH) were obtained from VWR (Ismaning, Germany) in analytical or purer grade. Anhydrous magnesium sulfate (anh. MgSO_4_) was provided by Sigma-Aldrich (Steinheim, Germany) in analytical grade. The readily prepared d-SPE tubes Supel™ QuE PSA tube (150 mg Supelclean™ PSA (primary secondary amine), 900 mg anh. MgSO_4_) were bought from Sigma-Aldrich (Steinheim, Germany). Other tested d-SPE tubes and bulk materials are detailed in the Electronic Supplementary Material (ESM).

### Analytical standards

Reference standards for AOH, AME, ATX I, AOH-3-G, AOH-9-G, AOH-3-S, AME-3-G, and AME-3-S were either isolated from fungal extracts or synthesized as described in the literature [17, 19]. Reference standards for the following substances were obtained from the respective sources in brackets: TEN and TeA (Merck, Darmstadt, Germany); DON, 3-AcDON, and Fus X (Coring System Diagnostix, Gernsheim Germany); T-2 (LGC Standards/Dr. Ehrenstorfer, Wesel, Germany); HT-2 and ZEN (Sigma-Aldrich, Steinheim, Germany); NIV, ENN A, and ENN B (Cayman Chemicals, MI, USA); ENN A1 and ENN B1 (Enzo Life Science, New South Wales, Australia). The stable isotope-labeled standards (ILS) [^2^H_4_]-AOH, [^2^H_4_]-AME, [^13^C_6_]-TeA, [^15^N_3_]-ENN A1, and [^13^C_4_]-T-2 were synthesized as reported previously [18, 20–23]. [^13^C_15_]-DON and [^13^C_17_]-3-AcDON were obtained from Libios (Vindry Sur Turdine, France), and DON-3-G, [^13^C_21_]-DON-3-G, and [^13^C_22_]-HT-2 from Biopure (Tulln, Austria).

### Preparation of stock solutions

All reference compounds were quantified using nuclear magnetic resonance (qNMR) measurements as described in the literature [24]. Stock solutions were prepared in ACN in a concentration range of 0.001 to 100 µg/mL. The stability of the standards was tested as reported in the literature [17]. According to this, quality control standards were used, and the concentrations were monitored by long-term observation of signal intensities. The standards were measured regularly to ensure the right concentration and instrument performance. The standards were stored at –20 °C between these measurements until further use.

### Grinding

A representative amount of the sample (at least 50 g) was ground with a laboratory mill (Grindomix GM 200, Retsch GmbH, Haan, Germany) to fine flour. The samples were stored at room temperature until analysis.

### Sample homogeneity

The workup was conducted in duplicates. If the relative standard deviation (RSD) of any measurement was above 20% for analytes quantified via SIDA and 40% for analytes quantified via matrix-matched calibration (MMC), the workup was repeated. However, the RSD was below 15% in most first workups.

### Final sample preparation

One gram of the homogenized finely ground sample was weighed into a 15-mL centrifuge tube. The sample was spiked with the internal standards, namely 10 µL of 1 µg/mL [^2^H_4_]-AOH, 50 µL of 0.01 µg/mL [^2^H_4_]-AME, 20 µL of 1 µg/mL [^13^C_6_,^15^N]-TeA, 50 µL of 1 µg/mL [^13^C_15_]-DON, 20 µL of 0.1 µg/mL [^13^C_17_]-3-AcDON, 50 µL of 1 µg/mL [^13^C_21_]-DON-3-G, 20 µL of 0.1 µg/mL [^13^C_4_]-T-2, 10 µL of 0.1 µg/mL [^13^C_22_]-HT-2, and 50 µL of 0.01 µg/mL [^15^N_3_]-ENN A1. After an equilibration period of at least 30 min, 10 mL of ACN/H_2_O (80/20, v/v) containing 1% formic acid (FA) was added to the sample, and the extraction tube was shaken for 60 min at 350 rpm on a horizontal shaker (Kombischüttler KL 2, Edmund Bühler GmbH, Hechingen, Germany). After centrifugation (Centrifuge 5810 R, Eppendorf AG, Hamburg, Germany) for 5 min at 3220 g, the supernatant was transferred to a 25-mL beaker. The remaining residue was extracted a second and third time with 5 mL ACN/H_2_O (80/20, v/v) and 5 mL of ACN/H_2_O (70/30, v/v), respectively, each containing 1% FA. The extraction time was 30 min each. Shaking and centrifugation were conducted as described above. After centrifugation, the supernatants were combined.

FA (200 µL) was added to the supernatant to give a total acid concentration of 2%. The supernatants were mixed with 1.8 g MgSO_4_ and 0.45 g NaCl in a 50-mL centrifuge tube for a QuEChERS-like clean-up. The tubes were shaken vigorously by hand for 60 s and then centrifuged at 3220 g for 10 min.

From the upper ACN phase, 10 mL was added to the d-SPE tube (150 mg Supelclean™ PSA, 900 mg anh. MgSO_4_). The tube was shaken vigorously by hand for 30 s, followed by shaking on a horizontal shaker at 350 rpm for 15 min. The d-SPE tube was centrifuged at 3220 g for 15 min. After centrifugation, 8.5 mL of the supernatant was transferred to a 15-mL centrifuge tube.

Afterward, 4 mL of the supernatant was transferred to a 4-mL vial and dried at 40 °C under a constant nitrogen stream. When the vial was almost empty, it was refilled with 4 mL supernatant and dried completely. The dried residue was reconstituted in 200 µL MeOH/H_2_O (6/4), transferred to 1.5-mL plastic microtubes, and frozen at − 20 °C for at least 30 min. The microtubes were centrifuged (Laborzentrifuge 2K15, Sigma, Osterode am Hartz, Germany) for 15 min at 13,201 g at 4 °C. The supernatant was membrane-filtered (PVDF, 0.2 µm) into 1.5-mL glass vials containing micro-inserts. Samples were stored at − 18 °C until analysis.

In order to assess the sample concentration in the final extract, we want to give a short overview calculation. For simplification, we expect all analytes to be present in the ACN phase, which will have a volume of about 15.5 mL in the final extract. We use exactly 8 mL of the extract for evaporation and reconstitute in a final volume of 200 µL. This leads us to 51.6% of the sample equivalent being present in the final extract.

### Workup optimization

Workup optimization was conducted in duplicates. Wheat flour from local supermarkets was spiked with a representative selection of mycotoxins. After complete evaporation of all solvents, the workup was performed as described.

#### Extraction

The extraction time and extraction solvents were tested in various combinations. Different ratios of ACN and H_2_O and the addition of FA were tested for the extraction solvent. Extraction time was tested for 30, 60, 90, and 120 min. For the second and third extraction steps, 15, 30, or 60 min was applied. The first extraction was conducted using 10 mL of extraction solvent, while for the second and third extraction, 5 mL was used. The solvents tested were ACN and H_2_O in the ratios 5/5, 6/4, 7/3, 8/2, and 84/16 (v/v), each without the addition of FA and with the addition of 0.1, 0.5, 1.0, 2.0, and 5.0% FA.

#### QuEChERS clean-up

The composition of the QuEChERS salts was varied during method optimization. The experiments were based on the originally described QuEChERS approaches used to calculate the added salt content [15, 25, 26]. Adding more of the salts did not improve the phase separation. Classic QuEChERS was conducted using 0.4 g anh. MgSO_4_ and 0.1 g NaCl per mL H_2_O in the extraction solvent. For the ammonium formate (NH_4_HCO_2_)–buffered variant, 0.4 g NH_4_HCO_2_, 0.1 g NaCl, and 0.4 g anh. MgSO_4_ were used per mL H_2_O. For the citrate-buffered variant, 0.4 g anh. MgSO_4_, 0.1 g NaCl, 0.1 g sodium citrate trihydrate, and 0.05 g sodium citrate sesquihydrate were used, respectively. In contrast, 0.4 g anh. MgSO_4_ and 0.1 g sodium acetate were used per mL H_2_O for the acetate-buffered approach. Before adding QuEChERS salts, the acidification by adding 200 µL FA was tested. It showed positive results for the extraction of TeA if 1% FA or less was used.

#### d-SPE clean-up

The application of various d-SPE sorbents was tested. The exact amounts are shown in the ESM. For d-SPE clean-up, 10 mL of the ACN layer was transferred into centrifuge tubes filled with the d-SPE sorbents. If the d-SPE combination was not commercially available in tubes, the sorbents were manually filled in centrifuge tubes. After shaking the d-SPE tubes for 15 min, the tubes were centrifuged, and 8 mL of the supernatant was taken out for drying. All d-SPE tubes and d-SPE bulk sorbents were bought from Sigma-Aldrich (Steinheim, Germany).

#### Injection solvent

The composition of the injection solvent was tested for different ratios and volumes of ACN, MeOH, and H_2_O. In detail, the volumetric ratios 9/1, 7/3, 6/4, 5/5, 4/6, 3/7, and 1/9 of MeOH/H_2_O and ACN/H_2_O were tested. The optimized workup approach was used for these trials, and the reconstitution solvent was changed accordingly.

### LC–MS/MS analysis

#### Chromatographic setup — UHPLC-column selection

To check the possibility of analyzing all mycotoxins in the optimized extract, different columns were tested using various solvent/column combinations. The columns tested are listed in detail in the ESM. The tested starting conditions involved H_2_O, ACN, and MeOH, without or with 0.1% and 1% addition of FA and acetic acid (AA), respectively. The addition of NH_4_HCO_2_ and ammonium acetate (NH_4_CH_3_CO_2_) was tested in 1.0, 2.0, and 5.0 mM concentrations at pH values of 8.0, 8.5, 8.7, and 9.0.

#### LC and gradient parameters

A Shimadzu Nexera X2 UHPLC system (Shimadzu, Kyoto, Japan) was used for liquid chromatography. Chromatographic separation was finally established on a Waters BEH C18 UHPLC column (Acquity BEH C18, 100 mm, 1.7 µm × 2.1 mm; Waters GmbH, Eschborn, Germany) for all toxins. The column was kept at 40 °C, and a 0.3 mL/min flow rate was used. A 5 mM NH_4_HCO_2_ solution in H_2_O at pH 9 was used for solvent A, and pure MeOH for solvent B. The pH was adjusted by using 25% ammonia solution. For better sensitivity, an injection volume of 10 µL was used. For obtaining a better peak shape for early eluting analytes, the co-injection of 40 µL H_2_O with NH_4_HCO_2_ at pH 9 was used (20 µL before and after the 10 µL sample, respectively). The binary gradient was programmed as follows: 0–2 min 5% B, then B was raised in 1 min to 18%. A slow increase was added within 2 min to 25% B. The concentration of B was then raised in 8 min to 90% and further raised to 99% B in 0.5 min. Finally, 99% B was held for 2 min. The concentration of solvent B was then brought back to 5% in 3.5 min and equilibrated for 5 min.

#### Source and MS parameters

The UHPLC system was coupled to a Shimadzu 8050 triple quadrupole mass spectrometer (Shimadzu Corporation, Kyoto, Japan). The following ion source parameters were used: heat block temperature 450 °C, interface temperature 350 °C, desolvation temperature 150 °C, interface voltage 3 kV for positive ionization and − 3 kV for negative ionization, drying gas flow 10 L/min, heating gas flow 10 L/min, nebulizing gas flow 3 L/min, collision-induced dissociation gas pressure 270 kPa. Polarity switching enabled simultaneous measurement in positive and negative electrospray ionization (ESI) mode in the same LC–MS/MS run. Different ionization polarities were necessary for increased analyte sensitivity. All measurements were conducted in multiple reaction monitoring (MRM) mode. All mass transitions used in the final method are shown in Table 1.

**Table 1:** LC–MS/MS parameters of the analyzed mycotoxins; Rt = retention time

Analyte	Measured ion	Precursor ion (m/z)	Product ions (m/z)	Q1 Pre bias (V)	CE (V)	Q3 Pre bias (V)	Rt (min)
AOH	[M – H]^–^	257.30	213.00/214.85	28.0/26.0	25.0/23.0	34.0/14.0	9.45
[^2^H_4_]-AOH	[M – H]^–^	261.30	217.00/218.85	28.0/26.0	25.0/23.0	34.0/14.0	9.45
AOH-3-G	[M – H]^–^	419.10	256.15/255.10	30.0/16.0	33.0/44.0	26.0/26.0	7.22
AOH-9-G	[M – H]^–^	419.10	256.15/255.10	30.0/16.0	33.0/44.0	26.0/26.0	8.82
AOH-3-S	[M – H]^–^	337.10	257.15/213.10	24.0/24.0	22.0/40.0	24.0/18.0	7.49
AOH-9-S	[M – H]^–^	337.10	257.15/213.10	24.0/24.0	22.0/40.0	24.0/18.0	12.76
AME	[M – H]^–^	271.25	256.00/255.10	14.0/34.0	23.0/28.0	10.0/26.0	12.76
[^2^H_4_]-AME	[M – H]^–^	275.25	260.00/259.10	14.0/34.0	23.0/28.0	10.0/26.0	11.35
AME-3-G	[M – H]^–^	433.30	270.20/271.20	16.0/12.0	33.0/26.0	18.0/20.0	11.20
AME-3-S	[M – H]^–^	351.20	271.20/256.15	12.0/12.0	23.0/35.0	26.0/24.0	10.84
ATX I	[M – H]^–^	351.20	315.00/333.05	26.0/26.0	17.0/12.0	18.0/36.0	11.20
TeA	[M – H]^–^	196.40	111.95/139.00	22.0/22.0	25.0/19.0	34.0/26.0	4.65
[^13^C_6_, ^15^N]-TeA	[M – H]^–^	203.40	112.95/142.00	22.0/22.0	25.0/19.0	34.0/26.0	4.65
TEN	[M – H]^–^	413.40	141.05/271.30	14.0/14.0	23.0/20.0	12.0/16.0	11.78
3-AcDON	[M + H]^+^	339.10	231.25/175.20	﻿ − 16.0/﻿ − 16.0	﻿− 13.0/﻿ − 25.0	﻿− 26.0/ ﻿− 20.0	8.73
[^13^C_17_]-3-AcDON	[M + H]^+^	356.10	245.25/186.20	﻿− 16.0/ ﻿− 16.0	﻿ − 13.0/ ﻿− 25.0	﻿ − 26.0/ ﻿− 20.0	8.73
BEA	[M + NH_4_]^+^	801.50	244.20/134.20	− 18.0/ − 18.0	− 33.0/ − 55.0	− 18.0/ − 14.0	14.50
DON	[M + H]^+^	297.15	249.20/231.15	− 14.0/ − 18.0	− 11.0/ − 12.0	− 18.0/ − 26.0	5.75
[^15^C_13_]-DON	[M + H]^+^	312.15	263.20/245.15	− 14.0/ − 18.0	− 11.0/ − 12.0	− 18.0/ − 26.0	5.75
DON-3-G	[M – H]^–^	457.30	427.05/255.35	22.0/16.0	17.0/27.0	44.0/30.0	6.05
[^13^C_21_]-DON-3-G	[M – H]^–^	478.15	447.05/261.30	22.0/16.0	17.0/27.0	44.0/30.0	6.05
ENN A1	[M + NH_4_]^+^	685.45	210.25/100.30	− 20.0/ − 16.0	− 29.0/ − 55.0	− 14.0/ − 22.0	14.70
[^15^N_3_]-ENN A1	[M + NH_4_]^+^	688.45	211.25/101.30	− 20.0/ − 16.0	− 29.0/ − 55.0	− 14.0/ − 22.0	14.70
ENN A	[M + NH_4_]^+^	699.45	210.20/100.30	− 16.0/ − 16.0	− 33.0/ − 55.0	− 22.0/ − 24.0	14.85
ENN B1	[M + NH_4_]^+^	671.35	196.20/210.25	− 16.0/ − 16.0	− 36.0/ − 32.0	− 14.0/ − 14.0	14.65
ENN B	[M + NH_4_]^+^	657.45	196.20/86.20	− 18.0/ − 26.0	− 32.0/ − 64.0	− 14.0/ − 18.0	14.30
Fus X	[M + H]^+^	372.15	355.25/337.20	− 18.0/ − 18.0	− 9.0/ − 13.0	− 18.0/ − 24.0	7.40
NIV	[M – H]^–^	311.20	281.15/191.20	22.0/12.0	11.0/21.0	16.0/22.0	4.65
T-2	[M + NH_4_]^+^	484.35	305.20/215.30	− 26.0/ − 28.0	− 15.0/ − 21.0	− 22.0/ − 24.0	12.13
[^13^C_4_]-T-2	[M + NH_4_]^+^	488.35	307.15/216.25	− 26.0/ − 28.0	− 15.0/ − 21.0	− 22.0/ − 24.0	12.13
HT-2	[M + NH_4_]^+^	442.20	263.25/215.15	− 22.0/ − 22.0	− 14.0/ − 14.0	− 10.0/ − 24.0	11.57
[^13^C_22_]-HT-2	[M + NH_4_]^+^	464.20	278.25/200.15	− 22.0/ − 22.0	− 14.0/ − 14.0	− 10.0/ − 24.0	11.57
ZEN	[M – H]^–^	413.40	141.05/271.30	14.0/14.0	23.0/20.0	12.0/16.0	12.37

#### Calibration and quantification

SIDA was used for all toxins where ILS were available, namely DON, DON-3-G, 3-AcDON, T-2, HT-2, and ENN A1 of the *Fusarium* toxins and AOH, AME, and TeA of the *Alternaria* toxins. ENN A, ENN B, ENN B1, and BEA were quantified with response curves using [^15^N_3_]-ENN A1 as a structurally similar internal standard (IS). The other toxins, namely NIV and ZEN of the *Fusarium* toxins and TEN, ATX I, AOH-3-G, AOH-9-G, AOH-3-S, AME-3-G, and AME-3-S of the *Alternaria* toxins, were quantified via MMC.

Response curves were prepared for those toxins for which internal standards were available. The curves were created by mixing analytes (A) with their respective standards (S) (either ILS or IS) in specific amounts to obtain molar ratios n(A)/n(S) ranging from 0.01 to 100 (1:100, 1:50, 1:25, 1:10, 1:5, 1:2, 1:1, 2:1, 5:1, 10:1, 25:1, 50:1, 100:1). The absolute amount of ILS or IS was kept constant. For TeA, AOH, AME, DON, 3-AcDON, DON-3-G, T-2, HT-2, and ENN A1, their respective isotopologues [^13^C_6_,^15^N]-TeA, [^2^H_4_]-AOH, [^2^H_4_]-AME, [^13^C_15_]-DON, [^13^C_17_]-3-AcDON, [^13^C_21_]-DON-3-G, [^13^C_4_]-T-2, [^13^C_22_]-HT-2, and [^15^N_3_]-ENN A1 were used as ILS. For ENN A, ENN B, ENN B1, and BEA, [^15^N_3_]-ENN A1 was used as IS for the whole group of depsipeptides. Following the LC–MS/MS measurement, the peak area ratios [A(A)/A(S)] were plotted against the corresponding molar ratios [n(A)/n(S)] to obtain a response function through linear regression. The applicability of linear regression was verified using Mandel’s fitting test [27].

MMC curves were measured using potato starch (Merck KGaA, Darmstadt, Germany) as a blank matrix to quantify all other toxins. To verify the applicability, 8–10 MMC points were analyzed for each toxin in different ranges expected to occur in real samples. We analyzed the following concentrations: 0.5–20 µg/kg (ATX I), 2–100 µg/kg (NIV), 0.1–5 µg/kg (TEN), 0.2–50 µg/kg (ZEN), 0.2–20 µg/kg (AOH-3-G), 0.2–20 µg/kg (AOH-9-G), 0.2–20 µg/kg (AOH-3-S), 1–20 µg/kg (AME-3-G), 0.2–20 µg/kg (AME-3-S), 2–100 µg/kg Fus X. The peak areas [A(A)] were plotted against the concentration of analytes [c(A)]. Calibration curves were calculated by linear regression, and the linear model was confirmed by Mandel’s fitting test [27].

The response and MMC curves were used to quantify the analytes in cereal samples. To compensate for day-to-day variation, a response mix containing all analytes and their ILS was included in each measurement batch. For MMC, three concentrations in the calibration range were worked up and included in every batch as quality control. Quantification of all samples was performed in duplicates and double injection. If the precision was unsatisfactory, repeated measurements or repeated workups were conducted.

### Method validation

#### LODs and LOQs

Limits of detection (LODs) and limits of quantification (LOQs) were determined according to the literature [28]. Mycotoxin-free potato starch (Merck KGaA, Darmstadt, Germany) was used as a blank matrix. The blank matrix was spiked in triplicate with the analytes and respective internal standards at four different concentration levels, starting at the estimated LOD value and going up to about ten times the estimated LOD. Details about spiking concentrations can be found in the ESM. All samples were worked up according to the optimized method.

#### Recovery

To evaluate the recovery, the blank matrix (potato starch) was spiked in triplicate in three to four concentrations that resembled realistic toxin concentrations in the real samples. Starting from a value near the LOQ, higher concentrations were used up to ten times the LOQ for most toxins and even more for TeA, 3-AcDON, and DON. In the ESM, the details of the spiking experiments are described. After the sample workup and LC–MS/MS analysis, the recovery percentage was calculated as the ratio of the quantified amount of toxin divided by the spiked concentration times 100.

#### Precision

The blank matrix was spiked in triplicate with all analytes and corresponding standards and was worked up according to the developed QuEChERS approach. For intra-day precision (repeatability condition of measurement), three samples were worked up on the same day (*n* = 3), and for inter-day precision (intermediate precision condition of measurement), three samples were worked up on three different days (*n* = 9), respectively. The measurement was conducted in triplicates in consecutive weeks. Inter-injection precision (*n*=10) was determined as the standard deviation of ten successive injections of a toxin mix containing all analytes and internal standards.

#### Analysis of certified reference material (CRM)

The CRM QCM Biopure “deoxynivalenol in wheat” was bought from Biopure (Tulln, Austria). It was used to test the trueness of the analyzed DON concentration.

### Analysis of commercial products

A wide variety of 136 samples were bought in supermarkets for analysis. The sample set consisted of a variety of cereals and cereal-based products, namely 12 wheat flours, 8 rye flours, 10 spelt flours, 58 rice varieties and products, 7 oat products, 4 millet products, 3 quinoa products, 3 buckwheat products, 2 maize flowers, and 1 amaranth package as well as 28 different cereal-based infant foods and snacks for young children.

### Data analysis

The software LabSolutions version 5.118 (Shimadzu, Kyoto, Japan) was used to integrate the peak areas. Analyte concentrations, response curves, and linearity were calculated using Microsoft Excel 2021 (Microsoft Co, Redmond, WA, USA).

Quantitative data were visualized using the R platform (version 4.4.0). The package tidyverse (2.0.0) [29] was used for data preprocessing and plotting. Color scales for figures were based on the packages RColorBrewer (1.1–3) [30].

## Results and discussion

A QuEChERS method for simultaneously analyzing 24 different *Fusarium* and *Alternaria* toxins was successfully developed and validated. Before the chosen modified QuEChERS setup was finally established, dilute-and-shoot, SPE, and QuEChERS approaches were tested. Finally, the QuEChERS workup emerged as the most promising in performance, sensitivity, cost, and time requirements.

### Workup optimization

#### QuEChERS approach

A preliminary screening of several clean-up variants revealed that simple dilute-and-shoot was the least sensitive option for most analytes. Moreover, a direct comparison of SPE and QuEChERS followed by SPE had no advantages over a simple QuEChERS procedure, with the SPE being more time-consuming as it requires a double solvent exchange. Therefore, the QuEChERS approach was extensively optimized to be competitive with our group’s previously developed SPE methods while including more analytes, being faster, and reducing the workup time and costs. The workup was inspired by other QuEChERS-based procedures [31], which showed the applicability of the QuEChERS approach for a wide variety of mycotoxins and complex matrices.

#### Extraction

**Fig. 2:**
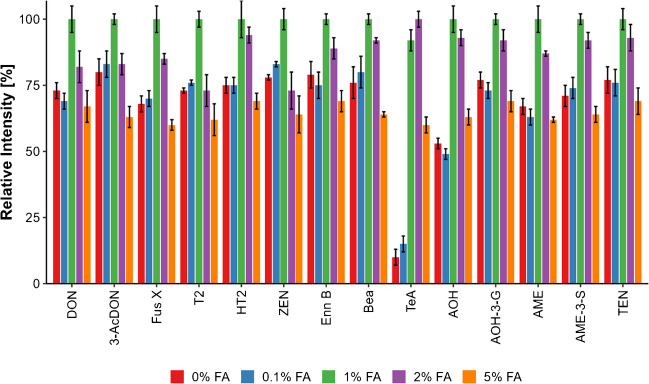
Comparison of extraction yields for important mycotoxins of the ACN/H_2_O 80/20 extraction mixture with increasing percentages of FA. The experiment was conducted using double determination and double injection. The concentrations used for spiking were as follows: DON (20 µg/kg), 3-AcDON (10 µg/kg), Fus X (50 µg/kg), T-2 (5 µg/kg), HT-2 (5 µg/kg), ZEN (5 µg/kg), ENN B (1 µg/kg), BEA (1 µg/kg), TeA (20 µg/kg), AOH (10 µg/kg), AOH-3-G (10 µg/kg), AME (1 µg/kg), AME-3-S (1 µg/kg), TEN (10 µg/kg)

Multiple mycotoxins are commonly extracted with different ratios of ACN and H_2_O, often in conjunction with acidification using FA or AA. Various ratios and volumes of those solvents were tested during the optimization process. The extraction time was varied for 30, 60, 90, and 120 min. Based on the extraction mixture of ACN and H_2_O described in the literature [17, 32], the FA concentration adjustment proved necessary. Concentrations below 1% FA showed a substantial loss in TeA and displayed reduced extraction efficiency for modified *Alternaria* toxins, while concentrations above 2% led to increased matrix coextraction (Fig. 2). For exhaustive analyte extraction, it was necessary to perform three consecutive extraction steps, with the first extraction taking 60 min and the second and third extraction taking 30 min each. The first consists of 10 mL ACN/H_2_O (80/20; v/v), the second of 5 mL ACN/H_2_O (80/20; v/v), and the third of 5 mL ACN/H_2_O (70/30; v/v), all containing 1% FA.

#### QuEChERS clean-up

To separate the matrix components and keep pH-sensitive analytes in the ACN phase during QuEChERS clean-up, the FA content was varied between 0 and 5%. Also, the three established variants of non-buffered, acetate-buffered, and citrate-buffered methods were tested [15, 25, 26], while the salt addition was based on the total H_2_O content of the final extraction solvent [25]. While all those methods were suitable for multi-mycotoxin workup, the approach using anh. MgSO_4_ and NaCl was finally applied, as no buffering was found to be necessary for the recovery of the analytes, and manually weighing only two components saves time. A surplus of QuEChERS salts did not lead to beneficial or adverse effects during the workup. Thus, the amount of salts used was adjusted to the amount of H_2_O left in the sample based on the initially published method [15]. To guarantee the transfer of TeA in the ACN phase, 1% FA was added before QuEChERS clean-up, leading to a final FA concentration of 2%.

#### d-SPE clean-up

**Fig. 3:**
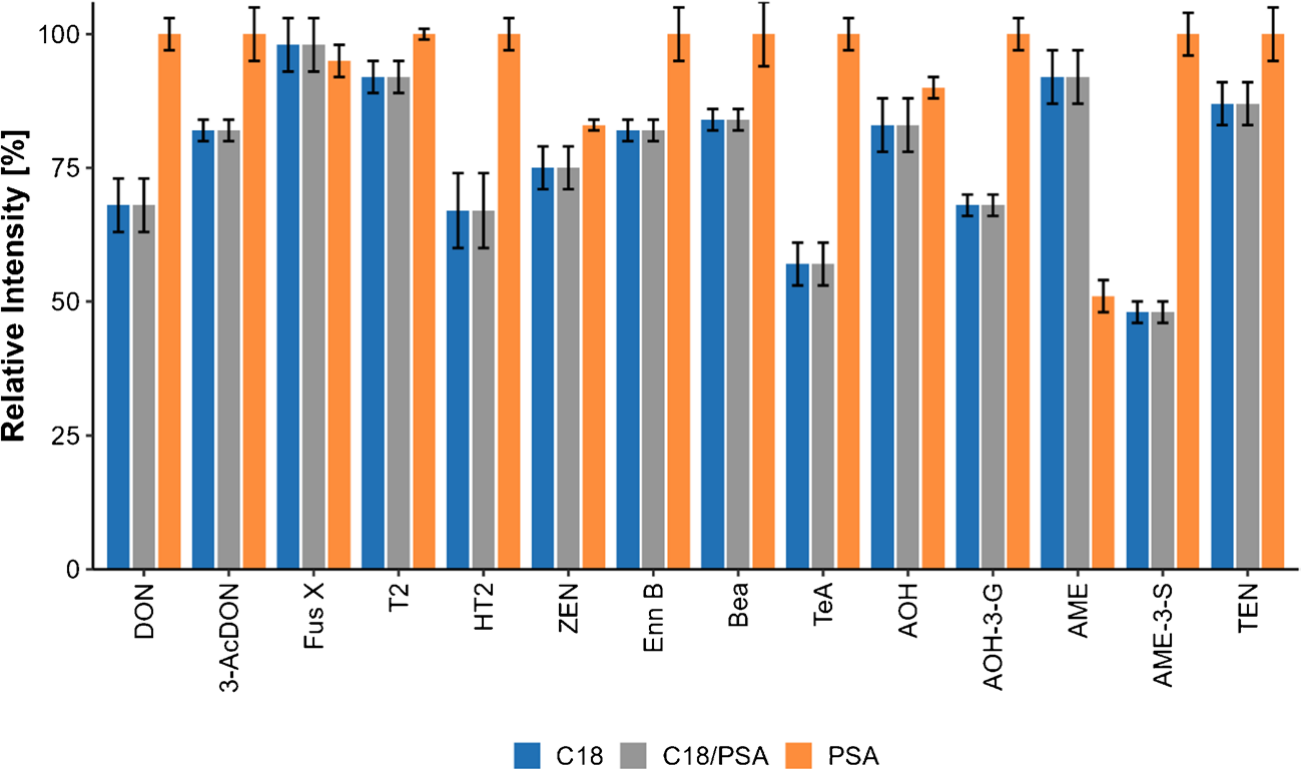
Comparison of recoveries for important mycotoxins when using different d-SPE clean-up materials. The experiment was conducted using double determination and double injection. The concentrations used for spiking were as follows: DON (20 µg/kg), 3-AcDON(10 µg/kg), Fus X (50 µg/kg), T-2 (5 µg/kg), HT-2 (5 µg/kg), ZEN (5 µg/kg), ENN B (1 µg/kg), BEA (1 µg/kg), TeA (20 µg/kg), AOH (10 µg/kg), AOH-3-G (10 µg/kg), AME (1 µg/kg), AME-3-S (1 µg/kg), TEN (10 µg/kg)

To further clean up the extract, the following d-SPE sorbents were tested: C18, PSA, Supelclean™ ENVI Carb™, and Supel™ QuE Z-SEP. Of these, PSA is the most commonly used *d*-SPE sorbent. In our case, it showed an excellent clean-up without any adverse effects. C18 is the second most used sorbent. It showed advantages for most toxins while decreasing the sensitivity of TeA, the ENNs, and BEA. ENVI-carb is based on graphitized carbon black and strongly removed different matrix compounds, but unfortunately, quantitatively removed AME and the ENNs as well. The effect of the adsorbents tested was similar to reports in the literature [33]. The acidification before d-SPE clean-up solved the problem of TeA loss, as known in the literature [34]. The decision favoring the sole usage of PSA and anh. MgSO_4_ compared to combining C18/PSA and anh. MgSO_4_ was made due to the better sensitivity for TeA and DON (Fig. 3).

#### Reconstitution solvent

A low volume of 200 µL revealed the best intensity and signal-to-noise ratio for all analytes, while matrix contamination was still acceptable. The low reconstitution volume is possible by the good clean-up achieved by QuEChERS and d-SPE. Ratios of 6/4 (v/v) for MeOH/H_2_O gave the best results for the re-solvation of analytes, matrix reduction, and peak shape. To prevent matrix precipitation during storage or at the autosampler temperature of 4 °C, the reconstituted extract was frozen for 30 min. Afterward, the sample was filtered with a PVDF filter before LC–MS/MS analysis. PVDF and PTFE filters were compared for the latter filtration. At the applied solvent ratio of 6/4 (v/v) MeOH/H_2_O, the PVDF filter showed better results and no specific analyte loss compared to the observations described in the literature [35].

### Development of the LC–MS/MS method

Concerning the expected occurrence and quantitative amounts of the mycotoxins in our study, a clear focus was set on the *Alternaria* toxins TeA as the major toxin of these fungi and the benzopyrones AOH and AME due to their toxicity. It is essential to get an insight into the occurrence of these *Alternaria* toxins and to obtain LODs and LOQs as low as possible to circumvent left-censored data for accurate exposure studies. Therefore, the development of the multi-method was targeted towards these aims, especially as TeA is not included in multi-methods in general, which is a general drawback we wanted to solve in our study.

#### Column selection

Selecting a suitable column was essential to achieve a reasonable separation of the analytes, especially the structural isomers. Different columns were initially tested based on availability and experience reported in the literature. Among those were various modified C18 columns, such as Acquity BEH C18 (Waters), Acquity CSH C18 (Waters), Acquity TSS T3 (Waters), Gemini® C18 (Phenomenex), HyperClone C18 (Phenomenex), Triart C18 (YMC), and Shim-pack Velox PFPP (Shimadzu), and others like biphenyl and phenylhexyl variants. Due to the number of different analytes with similar properties, it appeared straightforward to use modern UHPLC columns that allow for sharper peaks and better separation. It was quickly apparent that C18 columns showed the overall best separation results, as they are applicable to a wide range of analytes. The only disadvantage of the tested C18 columns was the inability to separate 3-AcDON from its isomer 15-AcDON. However, this disadvantage was accepted because 3-AcDON occurs more frequently in foods. After the first tests, the best results were achieved using three columns, namely the PFPP, BEH C18, and HSS T3 columns. The BEH C18 and HSS T3 columns showed promising results in direct comparison. Therefore, the chromatographically challenging molecule TeA that performs best in strong acidic or basic eluents determined the final column choice. As the latter two columns have a different pH working range, the final column selection was highly influenced by the choice of solvent. As the BEH C18 column is stable from pH 1 to 12, it was preferred and showed the best peak shape for TeA while still being compatible with all other analytes.

#### Solvents

To achieve the aim of separating all analytes within only one chromatographic run, the solvents required special attention. In particular, for the implementation of TeA into the method with a reasonably sharp peak, two options, according to literature and own experiments, are possible: the measurement either with 1% AA both in H_2_O and in ACN or the measurement with MeOH and H_2_O with NH_4_CH_3_CO_2_ at pH 9 [32, 36]. A range of pH values and buffer concentrations were tested for NH_4_CH_3_CO_2_ and NH_4_HCO_2_, and the best peak shape for TeA was obtained with 5 mM NH_4_HCO_2_ at pH 9. Additionally, NH_4_HCO_2_ favored the formation of NH_4_^+^ adducts in the ion source of the MS for better sensitivity of ENNs, BEA, T-2, and HT-2 without compromising the sensitivity of other toxins.

#### Gradient

**Fig. 4:**
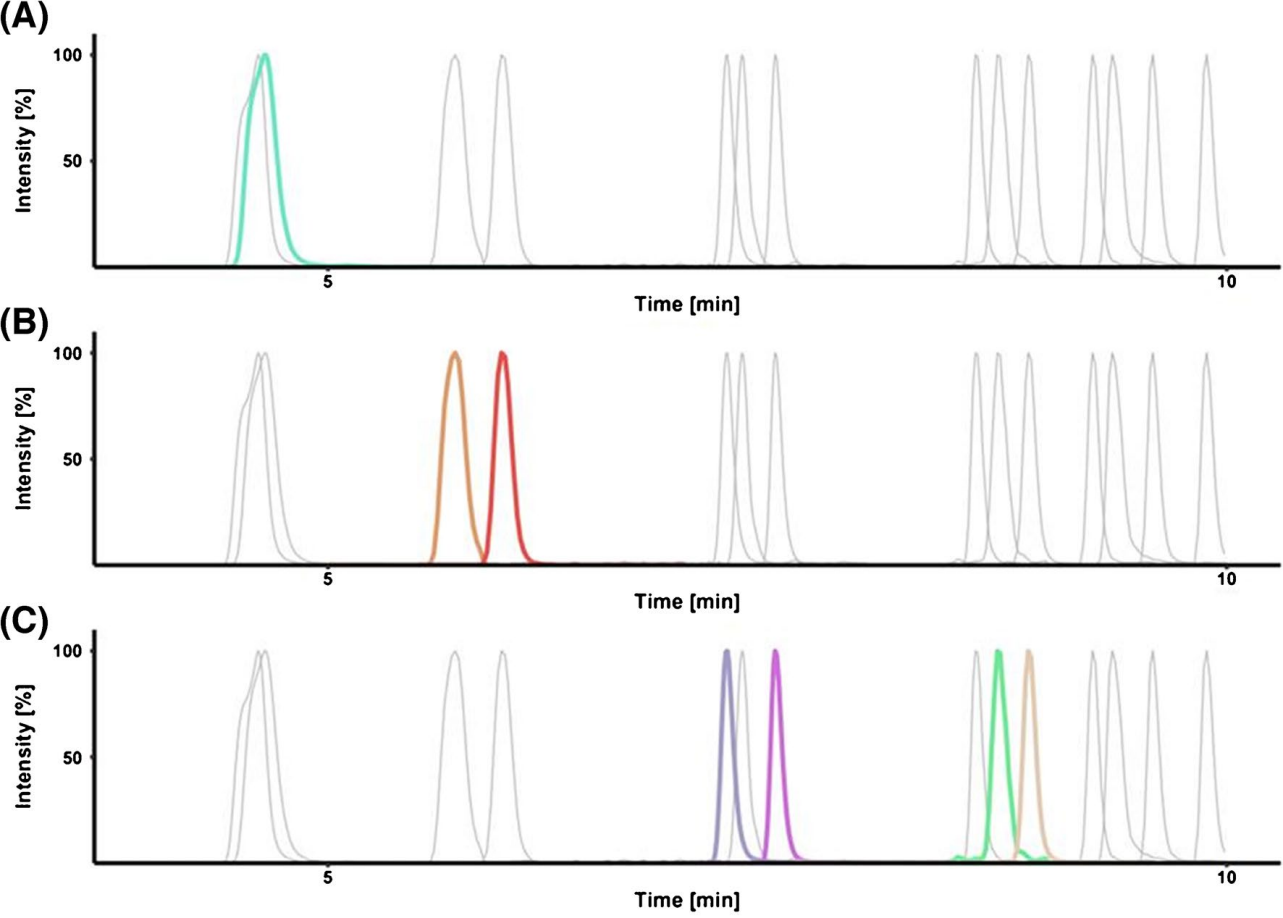
Excerpts of a multi-analyte chromatogram highlighting TeA (**A**), DON and DON-3-G (**B**), and the isomer pairs AOH-3-G/AOH-9-G and AOH-3-S/AOH-9-S (**C**). The concentrations used for the measurements were DON (0.1 µg/mL), DON-3-G (0.2 µg/mL), AOH-3-G (0.005 µg/mL), AOH-9-G (0.01 µg/mL), AOH-3-S (0.005 µg/mL), and AOH-9-S (0.005 µg/mL)

The flow rates were tested in the range between 0.2 and 0.4 mL/min, and a flow rate of 0.3 mL was elucidated as optimal as it allows sharp peaks while ensuring reduced pressure. Moreover, a column temperature of 40 °C gave the best performance. Although baseline separation for all analytes was not achievable, the specificity of MS/MS detection allowed us to quantify them separately. However, we had to compromise for separating 3-AcDON and 15-AcDON as the PFPP column capable of separating these isomers was omitted as it did not yield a reasonable peak shape for TeA (Fig. 4A). The alkaline mobile phase also allowed us to resolve the isomer pairs AOH-3-G/AOH-9-G and AOH-3-S/AOH-9-S (Fig. 4C) that are not differentiable by MS. Special attention had to be paid to the starting conditions: starting at 5% of the organic component B sharpened the peaks of TeA and NIV and in combination with the shallow gradient slope from 18 to 25% B allowed for the baseline separation of DON and DON-3-G (Fig. 4B). This is particularly important because DON-3-G shows in-source fragmentation to DON and might otherwise add to the DON signal. The other toxins did not require special attention, and the linear gradient achieved a sufficient resolution.

#### ESI polarity switching

**Fig. 5:**
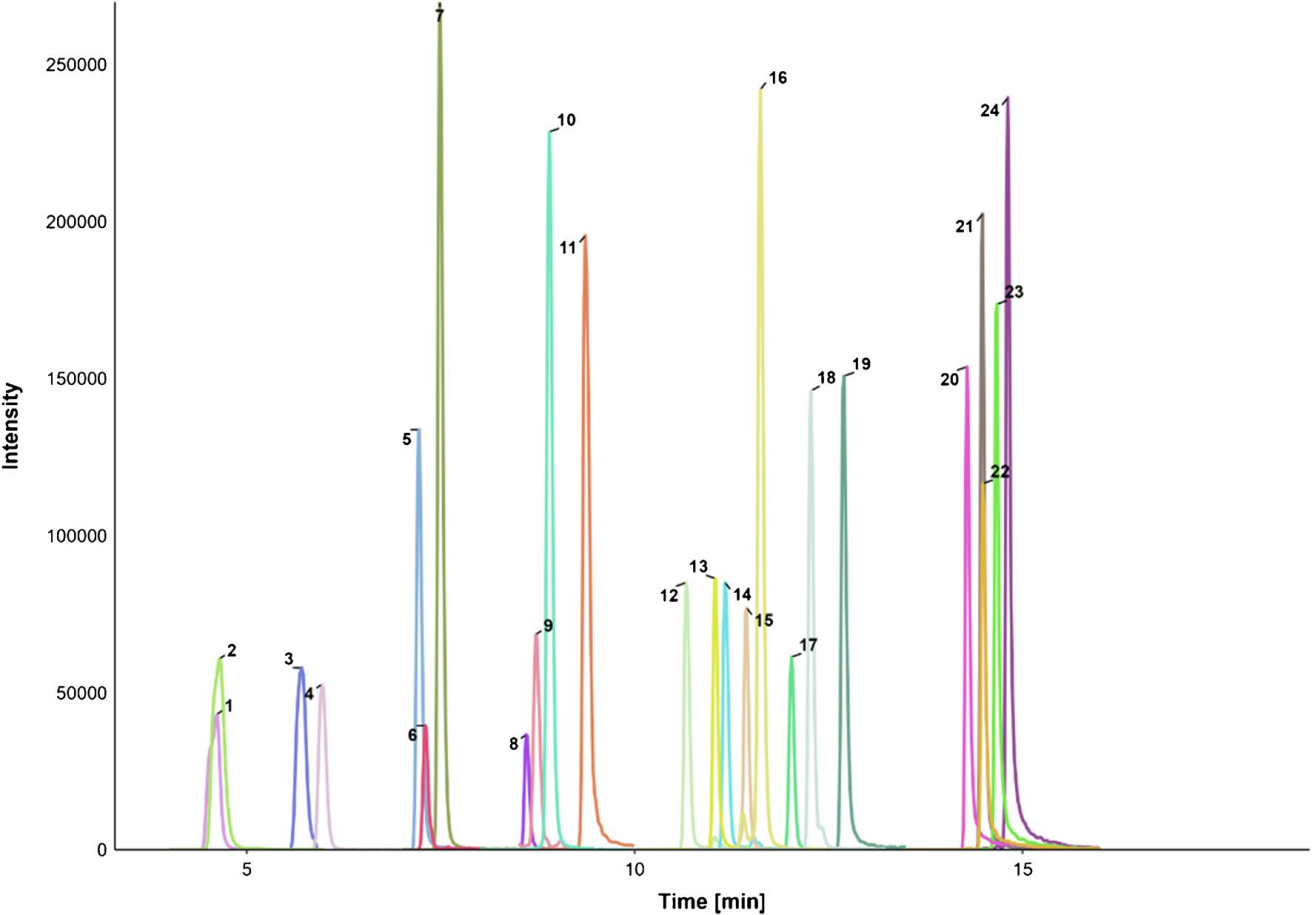
Full chromatogram of LC–MS/MS separation of the following mycotoxins in order of increasing retention time in the respective concentrations: NIV (1; 0.5 µg/mL), TeA (2; 0.2 µg/mL), DON (3; 0.1 µg/mL), DON-3-G (4; 0.2 µg/mL), AOH-3-G (5; 0.005 µg/mL), Fus X (6; 0.05 µg/mL), AOH-3-S (7; 0.005 µg/mL), 3-AcDON (8; 0.01 µg/mL), AOH-9-S (9; 0.005 µg/mL), AOH-9-G (10; 0.01 µg/mL), AOH (11; 0.03 µg/mL), AME-3-S (12; 0.0005 µg/mL), ATX I (13; 0.15 µg/mL), AME-3-G (14; 2 µg/mL), HT-2 (15; 0.02 µg/mL), TEN (16; 0.02 µg/mL), T-2 (17; 0.005 µg/mL), ZEN (18; 0.02 µg/mL), AME (19; 0.002 µg/mL), ENN B (20; 0.0004 µg/mL), BEA (21; 0.0004 µg/mL), ENN B1 (21; 0.0004 µg/mL), ENN A1 (23; 0.0004 µg/mL), ENN A (24; 0.0004 µg/mL)

During initial ESI tuning, it became apparent that some analytes could be detected more sensitively in the negative mode and others in the positive mode despite the variation of pH and mobile phase. This pointed to the need for polarity switching, which displayed similar sensitivity and stability as the singular ionization modes. With the implementation of this feature, we were able to reliably and sensitively measure 24 different mycotoxins, including their modifications in one method, as displayed in Fig. 5.

#### Source parameter optimization and polarity switching

Source optimization was conducted by manually modifying the desolvation line, heat block, and injector port temperature. Here, a quite low desolvation line temperature of 150 °C showed the best impact on the occurrence of NH_4_^+^ adducts while having a positive to no effect on the other toxins. The chosen heat block temperature of 450 °C and interface temperature of 350 °C displayed the highest intensity of analytes on average.

#### Comparison of the chromatographic performance

The newly developed method allows combining the two previously applied workup methods and four different LC–MS/MS methods previously used by our group in one LC–MS/MS run [17, 18, 37]. Analyzing *Alternaria* toxins under unusual basic conditions enables a high sensitivity for TeA. This is already described in the literature, but its compatibility with all other analytes and introducing *Fusarium* toxins in an *Alternaria*-focused method is a new feature of our method [20, 32, 34]. Moreover, optimizing all parameters allowed for low LOQs significantly below the current ML and IL in cereals and cereal-based foods (see next chapter).

However, some minor drawbacks of the new method also have to be mentioned: Two *Alternaria* toxins, ALTP and ATX II, are not quantifiable in contrast to one of our previous methods [17]. ATX II proved to be unstable during workup, and ALTP frequently overlapped with interfering matrix compounds. We also were not able to baseline separate 3-AcDON from its isomer 15-AcDON. We can detect the predominant isomer based on different mass transitions, but as their transitions overlap, we cannot quantify both simultaneously. The additional transitions for 15-AcDON were still included in the LC–MS/MS measurements, but we did not detect 15-AcDON in significant ratios in any of the 136 samples measured. This is reasonable, as *Fusarium* species usually only produce one major isomer. However, if a sample may contain a significant share of 15-AcDON, the respective extract could be measured with our previously published method using the PFPP stationary phase to separate the two isomers [37].

### Method validation

#### Calibration and quantification

Linearity of the SIDA response functions of the analytes AOH, AME, TeA, DON, DON-3-G, 3-AcDON, T-2, HT-2, ENN A, ENN A1, ENN B, ENN B1, and BEA in relation to their LIS was verified with Mandel’s fitting test [27]. The linear range encompassed molar ratios from 0.01 to 100 for all toxins except DON-3-G and ENN A1. For ENN A1 the linear range covered molar ratios n(A)/n(LIS) between 0.01 and 20. For DON-3-G, the range was deliberately reduced (0.1 to 50), as the respective LIS is quite expensive and added to the sample in relatively low amounts. However, this was no problem for DON-3-G, as ratios below 0.1 did not appear in the analyzed samples. For ENN A1, the range had to be reduced as the curve lacked linearity in ratios higher than 20. However, this is not critical as ENN A1 does not occur naturally in high amounts, and a ratio exceeding 20 did not seem probable from the perspective of the analyzed samples.

MMC curves were obtained for all samples by spiking the blank matrix with eight to ten concentrations. Linearity was again confirmed using Mandel’s fitting test [27]. The LOQ was used as the lowest spiking level, while the highest spiking level was at least ten times higher, resulting in the following calibration ranges: 0.1–30 µg/kg TEN, 1.0–20 µg/kg ATX I, 0.3–20 µg/kg AOH-3-S, 0.2–20 µg/kg AME-3-S, 0.2–20 µg/kg AOH-3-G, 0.2–20 µg/kg AOH-9-G, 1.0–20 µg/kg AME-3-G, 5.0–100 NIV, 2–100 µg/kg Fus X, and 0.2–50 g/kg ZEN.

The modified *Alternaria* toxin AOH-9-S was only included qualitatively in the method because the available amounts of this toxin were insufficient to generate an MMC for quantification.

### LODs and LOQs

LODs and LOQs were determined according to the literature [28]. Accordingly, an analyte-free blank matrix (potato starch) was spiked in four concentrations. The results are summarized in Table 2. The LODs ranged from 0.004 to 7.99 µg/kg. The ENNs and AME showed remarkably low LODs, which may be caused by a good ionization efficiency without many matrix interferences. In the case of the ENNs, especially the formed NH_4_^+^ adducts are sensitively detected in the MS. However, DON-3-G and NIV revealed high LODs as their ionization efficiency is worse, and they might dissolve partly in the H_2_O phase during QuEChERS clean-up. These trends have already been described in the literature [38]. Also, DON-3-G and AME-3-G showed reduced sensitivity, as both toxins tend towards in-source fragmentation, also already described in the literature [17, 32, 38].

**Table 2:** Limits of detection (LODs), limits of quantification (LOQs), relative standard deviation (RSD) values (precision), and recoveries for all toxins in starch as the blank matrix. Recovery values of each spiking level were determined as the mean value of three replicates in triple injection

Analyte	LOD (µg/kg)	LOQ (µg/kg)	Precision (RSD) (%)			Recovery (%)			
			Inter-injection (*n* = 10)	Intra-day (*n* = 3)	Inter-day ﻿(*n* = 9)	Level 1	Level 2	Level 3	Level 4
AOH^a^	0.20	0.79	3.6	5.2	5.5	98.5	95.5	97.7	95.1
AOH-3-G^c^	0.07	0.27	1.2	6.6	11.1	99.9	96.5	94.6	–
AOH-9-G^c^	0.16	0.50	3.9	4.5	12.7	98.2	105.4	99.9	–
AOH-3-S^c^	0.01	0.03	1.1	3.3	14.3	93.7	102.8	99.9	84.0
AME^a^	0.01	0.02	1.8	5.1	5.7	95.5	97.7	102.7	99.6
AME-3-G^c^	0.74	3.33	9.8	5.5	7.3	94.8	105.8	102.0	–
AME-3-S^c^	0.03	0.11	8.6	3.0	6.8	98.7	100.6	101.3	96.2
ATX I^c^	1.86	7.99	2.5	9.4	7.1	99.2	99.2	102.4	–
TeA^a^	0.14	0.50	1.2	5.9	6.1	101.4	94.8	95.5	101.0
TEN^c^	0.11	0.46	3.7	3.7	5.3	94.4	96.7	108.4	99.0
DON^a^	0.32	0.87	1.0	3.7	3.1	102.5	100.9	100.2	100.1
DON-3-G^a^	1.96	7.42	2.8	3.5	5.0	100.8	108.3	108.2	110.1
3-AcDON^a^	0.28	0.81	3.3	3.5	5.1	99.4	95.3	91.7	95.7
NIV^c^	3.16	11.8	4.5	8.2	7.1	101.3	99.4	100.6	–
Fus X^c^	0.52	2.37	3.4	7.5	12.1	110.2	96.8	102.4	–
ZEN^c^	0.08	0.30	3.2	7.5	10.2	90.8	102.3	99.8	–
T-2^a^	0.02	0.08	4.2	4.0	4.3	99.1	99.0	97.6	98.3
HT-2^a^	0.13	0.48	5.2	5.9	5.0	95.5	96.3	95.2	95.2
ENN A^b^	0.005	0.020	3.2	1.0	3.4	103.5	100.6	92.4	94.1
ENN A1^a^	0.004	0.013	3.1	5.1	5.8	102.8	102.5	107.9	103.7
ENN B^b^	0.005	0.021	3.0	1.7	3.6	107.8	103.9	101.4	99.9
ENN B1^b^	0.006	0.020	2.6	8.6	8.3	95.0	99.1	102.8	103.4
BEA^b^	0.006	0.020	3.5	3.7	5.8	102.8	105.6	94.9	97.0

^a^SIDA; ^b^IS [^15^N_3_]-ENN A1; ^c^MMC

The determined LODs and LOQs are comparable to our group’s previously developed single-species methods [17, 18], which we attempted to combine in the present study. The LOQs of the present method proved to be lower or similar for most analytes. Only ATX I showed a reduced sensitivity, which might be caused by the alkaline measuring conditions and high matrix-generated noise.

Our method displays competitive sensitivity compared to other multi-mycotoxin methods for a similar set of analytes. Setting a priority on TeA proved beneficial as the LOQs obtained were lower than those reported for other multi-methods [38, 39]. The sensitivity for the other *Alternaria* toxins was good and similar to those reported in the literature [17, 32, 38].

Sensitivity for *Fusarium* toxins that form NH_4_^+^ adducts like T-2, HT-2, ENN A, ENN A1, ENN B1, ENN B, and BEA was better than in other methods [18, 38, 40]. Moreover, the LOQs for DON, NIV, 3-AcDON, Fus X, and ZEN were low and in a similar range compared to methods in the literature. The LOQ of DON-3-G was slightly higher but still sufficiently low [38]. Overall, the method proved to be a sensitive quantification approach for all analytes.

#### Recovery

The recovery was determined by spiking every analyte in triplicates at three to four concentrations in the blank matrix. The lowest concentration was at the LOQ, while for the highest concentration, a reasonably high amount was chosen to establish the working range for the concentrations to be expected in the samples. As can be seen from Table 2, recoveries were between 84.0 and 108.3% for all analytes and thus met the criteria for recovery, staying between 70 and 120% [28]. Recoveries of around 100% are to be expected for all analytes determined by SIDA, but also for the other quantification methods, our results proved to be very satisfactory.

#### Precision

Intra-day, inter-day, and inter-injection precision were determined by calculating the RSD of every analyte after a defined number of repeated measurements. Inter-day precision was evaluated by preparing one sample in triplicate on the same day. Inter-day precision was generated by analyzing one sample in triplicates weekly for three weeks. Inter-injection precision was calculated after 10 times repeatedly injecting a toxin mix in solvent (MeOH/H_2_O, v/v) containing all analytes. Inter-injection RSD was between 1.2 and 9.8%, thus showing the stability of the system for most analytes. The relatively high variations for AME-3-S and AME-3-G might be caused by in-source fragmentation of the analytes. All obtained precisions are shown in Table 2.

#### Trueness

A CRM was used to prove the trueness of the method. However, the availability of CRMs with suitable analyte/matrix combinations was scarce. We decided to use a CRM of DON in wheat because this combination was predominant in our analyses of real samples. The measured DON content of 840 ± 67 µg/kg was well in line with the reference value of 825 ± 248 µg/kg.

### Sample analysis — application to samples

#### Wheat and pseudocereals

At first, we applied the combined *Fusarium* and *Alternaria* method to 50 classical baking and food grain products such as flours of wheat, rye, and spelt, along with oats and oat flakes, millet, and maize flours. We also analyzed the pseudocereals quinoa, amaranth, and buckwheat (Table 3). As expected, the bread cereal flours were dominated by DON with a maximum content of 200 µg/kg in wheat flour. Interestingly, the plant conjugate DON-3-G was only detected in the wheat samples, although spelt or rye samples also revealed significant contents of DON (see ESM). The ratio of DON-3-G to DON was 15% in wheat samples. The other trichothecenes 3-AcDON, NIV, T-2, and HT-2 were found sporadically and only in low concentrations in these cereal products. When comparing the different groups of cereals, clustering of T-2 and HT-2 in oat samples was observed, whereas the highest content of 6.6 µg/kg T-2 was found in a millet sample. Regarding ZEN, we found this toxin in 70% of all samples outlined before, with a maximum content of 3.2 µg/kg in a corn flour sample. ENNs and BEA were also found frequently in all of these samples but with contents below 15 µg/kg. No sample exceeded the current ML for these mycotoxins in the EU [3].

**Table 3:** *Fusarium* and *Alternaria* toxin contents in 50 classical baking and food grain products

	Positive samples (number/percent of ﻿all samples)	Mean of all positive samples﻿ (µg/kg)	Maximum content (µg/kg)
DON	37/74%	46.3	200
DON-3-G	12/24%	11.5	26
3-AcDON	3/6%	0.67	1.45
NIV	1/2%	3.16	3.16
HT-2	8/10%	1.28	5.99
T-2	11/11%	0.98	6.63
ZEN	35/70%	0.66	3.21
ENN A	29/58%	0.03	0.27
ENN A1	43/86%	0.16	1.08
ENN B	47/94%	3.96	14.2
ENN B1	45/90%	1.35	13.8
BEA	40/80%	0.03	0.18
TeA	38/76%	62.0	842
TEN	41/82%	4.00	17.7
AOH	13/26%	0.61	2.51
AOH-3-S	13/26%	0.09	0.33
AME	17/34%	0.58	2.25
AME-3-S	18/36%	0.14	0.32
ATX I	1/2%	1.86	1.86

Of the *Alternaria* toxins, the most prevalent compound, TeA, showed an interesting distribution. The wheat samples were hardly affected, but the other cereal samples were frequently contaminated and showed partly significant contents. The highest amount was found in a millet sample, which was not surprising when considering our previous reports on this cereal being particularly susceptible to TeA contamination [41, 42]. TEN was also frequently found among the other *Alternaria* toxins, but at minor contents, mostly below 10 µg/kg. AOH and AME were found sporadically, with no preference for a specific cereal. The respective sulfate conjugates were often found parallel with the two benzopyrones.

The pseudocereals quinoa, amaranth, and buckwheat showed minor contaminations with the mycotoxins under study except for TeA, for which most of these samples also revealed detectable contents. To the best of our knowledge, this is the first report of contaminations of DON, T-2, HT-2, AME, AME-3-S, and AOH-3-S in buckwheat samples. In quinoa and amaranth, we could only detect minor ENNs and BEA contamination along with TeA, TEN, AME, and AME-3-S.

There have been numerous reports on *Fusarium* and *Alternaria* mycotoxin contamination in cereal products. A comparison with literature data is quite difficult due to the dependence of the mycotoxin occurrence on the respective climatic and geographic growth conditions. Therefore, the following literature discussion is based on European Food Safety Authority (EFSA) opinions and comparisons to data from Germany.

The latest review published by EFSA on DON and its modified forms [43] stated a maximum DON content in European grains intended for human consumption of over 20 mg/kg from Finland, which was deemed unusually high compared to other data. The other contents from European countries peaked at 4130 µg/kg DON and 1070 µg/kg DON-3-G. Compared to our results, the higher content is not unexpected as complete grains generally contain more outer parts, which are usually more contaminated with mycotoxins. The ratio between DON-3-G to DON was reported to average around 0.2 [43], which is slightly higher than our results.

*Fusarium* toxins in German wheat flours were already assessed in 2002 [44]. We could confirm the reported ubiquitous contamination frequency with DON. However, the mean DON content of 290 µg/kg reported by the latter authors exceeded our mean of the wheat samples of 78.8 µg/kg by a factor of almost three. The reported occurrence and level of the other *Fusarium* toxins 15-AcDON, 3-AcDON, NIV, T-2, HT-2, and ZEN [44] were similar to our results.

The recent EFSA review on *Alternaria* toxins [45] revealed in wheat or grain milling products mean concentrations of 3.9, 0.7, 27.6, and 2.1 µg/kg for AOH, AME, TeA, and TEN, respectively. For AOH, the latter data are significantly higher than our results, whereas the AME data are quite similar. The low occurrence of TeA in the wheat flours of our study was unexpected and somehow contradictory to the results reported in the literature [45]. Similarly, our mean value for TEN of 0.6 µg/kg is also below the reported values.

Reports on *Fusarium* and *Alternaria* toxin occurrence in pseudocereals are rare. One could not detect any DON, 15-AcDON, 3-AcDON, NIV, T-2, HT-2, T-2 tetraol, and ZEN in the three pseudocereals we also analyzed [46]. EFSA reported only values for buckwheat [45]. We could not detect any AOH, whereas AME showed a maximum content of 1.7 µg/kg for one of the three samples we analyzed. These results were contradictory to the latter reports as these revealed mean contents of 30.5 and 10.6 µg/kg for AOH and AME, respectively, in buckwheat [45]. For TeA, we found contents of 118 and 22 µg/kg in the two positive samples, which is in good accordance with the latter authors. For TEN, we found only one positive sample of about 1 µg/kg in buckwheat samples, which does not differ from the mean content of 1.3 µg/kg in buckwheat milling products reported by EFSA [45].

#### Rice and rice products

The next type of cereals we looked at were rice grains, of which we analyzed an extended variety of 58 samples, including white, brown, fragrant, and organically grown rice (Table 4). As to be expected, the occurrence of the trichothecenes was very minor. Only about 40% of the analyzed samples contained DON at a mean of 33 µg/kg in the positive samples. The only exception was one long corn rice showing a DON content of 525 µg/kg. Our results showed somewhat lower contents than data in the literature that reported a mean DON content of 107 µg/kg in German samples [47] and 139 μg/kg in Korean samples [48]. The latter authors also reported a mean of 18.9 μg/kg for Nigerian samples, which aligns with our results. In the EU regulation [3], there are no legal limits for DON in rice, but our results show that rice still has to be screened for this toxin.

**Table 4:** *Fusarium* and *Alternaria* toxin contents in 58 rice samples

	Positive samples (number/percent of all samples)	Mean of all positive samples (µg/kg)	Maximum content (µg/kg)
DON	21/36%	33.4	525
3-AcDON	1/2%	0.28	0.28
HT-2	2/3%	0.18	0.22
T-2	10/17%	0.40	1.46
ZEN	26/45%	1.00	3.39
ENN A	4/7%	0.00	0.01
ENN A1	2/3%	0.07	0.10
ENN B	9/16%	0.46	3.21
ENN B1	7/12%	0.12	0.64
BEA	41/71%	1.04	17.2
TeA	55/95%	48.8	842
TEN	40/69%	1.58	9.36
AOH	11/19%	0.66	3.58
AOH-3-S	7/12%	0.13	0.47
AME	37/64%	0.36	3.14
AME-3-S	36/62%	0.19	0.68
ATX I	1/2%	1.86	1.86

Similarly to DON, half of all rice samples were contaminated with ZEN at a slightly higher mean concentration around 1 µg/kg compared to the other cereals and a similar maximum content of 3.4 µg/kg. Thus, the ZEN contamination in rice was lower than published by EFSA [49], where a 2.0 – 3.7 μg/kg mean content range was reported.

The distribution of the depsipeptides was interesting, as the contamination with ENNs was lower than for the other cereals. Again, the contamination of ENNs was much lower than stated by EFSA [50], where a mean concentration range for the sum of ENNs of 20.6 - 21.3 μg/kg was reported for grains for human consumption. However, the BEA content was significantly higher at an incidence of about 70%, with a mean content of about 1 µg/kg and a maximum of more than 17 µg/kg in one particular black rice sample. This concentration is even higher than the maximum concentration of 11.7 μg/kg for grains for human consumption given by EFSA [50]. As the only other black rice sample did not contain any BEA and no other report on BEA content in black rice could be found, it is unclear if this high concentration of BEA is exclusive to black rice samples. However, all other toxin contents were very similar to those reported by EFSA [50].

Similarly, as the other cereals, the rice samples were also frequently contaminated with the *Alternaria* toxin TeA at a similar mean content of 62 µg/kg and a maximum content exceeding 800 µg/kg. This is significantly higher than reported by EFSA, which found a mean content in rice of 22 µg/kg [45]. The contamination with TEN was also similar to the other cereals, with the toxin being quantifiable in about 70% of the samples. The distribution of the content of the benzopyrones and their modifications was also interesting as almost exclusively only AME and AME-3-sulfate were quantifiable in about 60% of the samples with a frequent co-occurrence of AME and its sulfate. In total, no clustering of mycotoxins was apparent when comparing the types of rice analyzed.

#### Infant foods

In the last set of samples, we analyzed 28 cereal-based infant foods, including some rice waffles for infants (Table 5). For DON and ZEN, none of the samples exceeded the upcoming EU ML of 150 μg/kg for DON [51]. Moreover, none of the modified forms of DON was detected, which was consistent with previous reports [52].

**Table 5:** *Fusarium* and *Alternaria* toxin contents in 28 cereal-based infant foods

	Positive samples (number/percent of all samples)	Mean of all positive samples (µg/kg)	Maximum content (µg/kg)
DON	9/32%	5.14	17.8
3-AcDON	1/4%	0.28	0.28
HT-2	2/3%	1.34	1.82
T-2	2/17%	0.18	0.34
ZEN	1/4%	0.08	0.08
ENN A	6/21%	0.06	0.22
ENN A1	12/43%	0.09	0.46
ENN B	14/50%	0.82	3.43
ENN B1	15/54%	0.27	1.25
BEA	24/86%	0.31	4.27
TeA	25/89%	49.0	240
TEN	14/50%	3.06	15.8
AOH	1/4%	0.20	0.20
AOH-3-G	1/4%	1.31	1.31
AME	13/46%	0.26	0.69
AME-3-S	7/25%	0.08	0.20

Moreover, none of the samples exceeded the newly introduced 10 μg/kg ML for the sum of T-2 and HT-2 [8]. T-2 was only observed once, but the content level was lower than the LOQ. This limited occurrence and lower content levels are consistent with other studies [53, 54].

None of the samples exceeded the EU ML of 20 μg/kg for ZEN [3]. Compared to other studies, our results were either slightly elevated [52] or lower [55]. Noteworthy is the higher occurrence rate for ZEN than reported in the literature [53], in which a 3% occurrence rate in processed cereal-based infant foods was described compared to our rate of 9%. This could be because our technique is more sensitive than the latter study, as our LOQ is 0.3 μg/kg in contrast to the LOQ of 0.6 μg/kg of the latter authors.

The contamination with ENNs occurred more frequently in infant foods than in rice samples. Occurrence rates of 35%, 52%, 57%, and 74% could be found for ENN A, ENN A1, ENN B, and ENN B1 in infant foods, respectively, compared to 12%, 9%, 9%, and 24% for the rice samples. The occurrence and contents of BEA were slightly higher than those of the ENNs but not as pronounced as in the rice products.

Compared to the other toxins, TeA had the highest content levels and highest occurrence rate in infant foods, which is comparable to other reports [41, 52]. TeA content levels for infant foods were lower than reported in the recent EFSA update on *Alternaria* toxins [45], where a mean of almost 500 µg/kg was reported for foods for infants and small children compared to our mean of about 50 μg/kg. Moreover, content levels found in our study were lower than those for millet samples in our previous report [42]. Similar to one of our prior studies [41], contamination levels of AOH were relatively low, with none of the samples exceeding the LOQ. AOH-3-G was found only once throughout the analysis in an infant food made from buckwheat. As mentioned above, pure buckwheat samples did not contain AOH-3-G. Thus, this content is not representative. However, 12 out of 23 samples (52%) contained AME, which differs from the literature that did not detect any AME [52]. This, again, could be due to our technique being more sensitive as we had an LOQ of 0.2 μg/kg compared to 1.0 μg/kg of the latter study. Moreover, the mean AME content was lower than reported in the literature [45, 53, 56, 57]. Half of the AME samples also contained the modified form AME-3-S, and as the AME-3-S content is often at the same level as the AME content, it is expected to significantly add to the total AME exposure. Currently, for screening purposes, the EC has set ILs of 2 µg/kg for AOH and AME, respectively, and 500 μg/kg for TeA in cereal-based infant food [11] following our first evaluation [58]. However, none of the samples exceeded these limits. Nevertheless, we found some of the raw materials for infant food, like rice or millet, exceeded these values, and modified toxins like the sulfates of AOH and AME contributed to the overall exposure. This still highlights the need for further screening infant foods for *Alternaria* toxins, including the modified forms reported here.

Out of our sample set, only three infant foods contained dried milk powder. Although we did not observe deviating performance during sample preparation, it should be noted that milk powder can precipitate in the presence of a high concentration of organic solvent, leading to analyte losses. The addition of internal standards prior to the addition of extraction solvent is crucial to obtain reliable results.

## Conclusion

The developed workup procedure provides a generally suitable approach for analyzing mycotoxins. It allows the analysis of 24 different *Alternaria* and *Fusarium* toxins while including their modified forms. The developed LC–MS/MS method offers a good opportunity to analyze all analyzed mycotoxins in one 23 min LC–MS/MS run.

The method was successfully validated and showed a high sensitivity for most analytes while providing sufficient sensitivity to detect mycotoxins below their regulatory limits. The method was applied in routine analysis to various cereal-based samples displaying the frequent co-occurrence of *Fusarium* and *Alternaria* toxins. On average, the contamination level was relatively low for Fusarium toxins, but the ENNs and BEA were found in almost all samples, yet in low amounts. The contamination of *Alternaria* toxins was relatively low, but the modified variations AOH-3-S and AME-3-S were frequently detected and appeared at levels similar to those of the unmodified toxins. Therefore, we conclude that they should be implemented in routine methods for a comprehensive risk assessment of *Alternaria* toxins.

Of the 136 analyzed samples, only 4 were completely free of the mycotoxins under study. All four of those samples were cereal-based infant foods. In general, the maximum toxin content in infant foods was lower compared to other cereals, being well below regulatory limits in all tested samples.

Our results revealed a mycotoxin contamination in over 95% of samples, which aligns with current reviews on the increasing co-occurrence and number of known mycotoxins [59].

The QuEChERS approach proved to be a robust, reliable, and cheap workup method for multi-mycotoxin analysis that promises great compatibility for many analytes over a vast range of polarities and matrices. Therefore, it is most likely that many other mycotoxins, like aflatoxins and ochratoxins, may be implemented in the workup procedure.

## Supplementary Information

Below is the link to the electronic supplementary material.

Supplementary file1 (DOCX 43 KB)

Supplementary file2 (XLSX 26 KB)

## Data Availability

The datasets generated during and analyzed during the current study are available from the corresponding author upon reasonable request.
