# Supplementary material for: Development of a high-throughput UHPLC-MS/MS method for the analysis of Fusarium and Alternaria toxins in cereals and cereal-based food
Source: Anal Bioanal Chem. 2024 Sep 2;416(26):5619–37. doi: 10.1007/s00216-024-05486-4 (PMC11493838; doi:10.1007/s00216-024-05486-4)
Supplement: Supplementary file 1 — Supplementary file1 (DOCX 43 KB) [file 216_2024_5486_MOESM1_ESM.docx]

**Electronic Supplementary Material**

1. **Tested d-SPE combinations**

| Name | PSA  [mg] | C18  [mg] | ENVI carb [mg] | Z-SEP  [mg] | MgSO_4_  [mg] |
| --- | --- | --- | --- | --- | --- |
| Supel^TM^ PSA/C18 Tube | 150 | 150 | / | / | 900 |
| Supel^TM^ PSA/C18 Tube | 400 | 400 | / | / | 1200 |
| Supel^TM^ PSA | 150 | / | / | / | 900 |
| Supel^TM^ PSA | 400 | / | / | / | 1200 |
| Supel^TM^ PSA/ENVI-Carv^TM^ | 150 | / | 15 | / | 900 |
| Supel^TM^ Z-SEP/C18 | / | 300 | / | 120 | / |
| Supel^TM^ PSA/C18 Tube | 50 | 50 | / | / | 900 |
| Supelclean^TM^ PSA | 50 | / | / | / | 900 |
| Discovery®C18 | / | 50 | / | / | 900 |
| Supelclean^TM^ PSA/ Discovery®C18 | 100 | 100 | / | / | 900 |
| Supelclean^TM^ PSA | 100 | / | / | / | 900 |
| Discovery®C18 | / | 100 | / | / | 900 |

1. **Tested LC-MS/MS columns**

| Column | Stationary  phase | Particle  size [µm] | Pore  size  (A) | Length  [mm] | Internal  diameter  [mm] |
| --- | --- | --- | --- | --- | --- |
| Phenomenex Hyperclone | C18 | 3 | 130 | 100 | 2.1 |
| Phenomenex Gemini | C18 | 3 | 110 | 100 | 2.1 |
| Shimadzu Shim-pack Velox | PFPP | 2.7 | 90 | 100 | 2.1 |
| Restek Raptor | C18 | 2.7 | 90 | 100 | 2.1 |
| Phenomenex Kinetex | Phenylhexyl | 2.6 | 100 | 100 | 2.1 |
| Phenomenex Kinetex | Biphenyl | 2.6 | 100 | 100 | 2.1 |
| Waters Acquity BEH | C18 | 1.7 | 130 | 100 | 2.1 |
| Waters Acquity BEH Shield RP 18 | C18 | 1.7 | 130 | 150 | 2.1 |
| Waters Acquity HSS T3 | C18 | 1.8 | 100 | 100 | 2.1 |
| Waters Acquity CSH | C18 | 1.7 | 130 | 150 | 2.1 |
| Waters Acquity BEH | Phenylhexyl | 1.7 | 130 | 150 | 2.1 |
| YMC Triart | C18 | 1.9 | 120 | 150 | 2.1 |

1. **SIDA Spiking for all analytes**

| Analyte | Internal Standard | Spike Level [µg/kg] |
| --- | --- | --- |
| AOH | [^2^H_4_]-AOH | 10 |
| AME | [^2^H_4_]-AME | 0.5 |
| TeA | [^13^C_6_, ^15^N] | 50 |
| DON | [^13^C_15_]-DON | 50 |
| 3-AcDON | [^13^C_17_]-3AcDON | 2 |
| DON-3-G | [^13^C_21_]-DON-3-G | 50 |
| T2-Toxin | [^13^C_4_]-T2 Toxin | 2 |
| HT2-Toxin | [^13^C_22_]-HT2 Toxin | 10 |
| ENN A | [^15^N_3_]-ENN A1 | 1 |
| ENN A1 | [^15^N_3_]-ENN A1 | 1 |
| ENN B | [^15^N_3_]-ENN A1 | 1 |
| ENN B1 | [^15^N_3_]-ENN A1 | 1 |
| Bea | [^15^N_3_]-ENN A1 | 1 |

1. **Equations of the Response Curves**

| Analyte | Equation | Linear Range n(a)/n(IS) |
| --- | --- | --- |
| AOH/[^2^H_4_]-AOH | 1.1578 + 0.0054 | 0.01 - 100 |
| AME/[^2^H_4_]-AME | 2.1756 + 0.0124 | 0.01 - 100 |
| TeA/[^13^C_6_, ^15^N]-Tea | 0.821 + 0.0005 | 0.01 - 100 |
| DON/[^13^C_15_]-DON | 1.0646 + 0.0005 | 0.01 - 100 |
| 3-AcDON/[^13^C_17_]-3AcDON | 1.4798 + 0.0009 | 0.01 - 100 |
| DON-3-G/[^13^C_21_]-DON-3-G | 1.1498 + 0.0152 | 0.02 - 50 |
| T2-Toxin/[^13^C_4_]-T2 Toxin | 1.2463 – 0.0001 | 0.01 - 100 |
| HT2-Toxin/[^13^C_22_]-HT2 Toxin | 0.8854 + 0.0220 | 0.01 - 100 |
| ENN A/[^15^N_3_]-ENN A1 | 1.8937 + 0.0006 | 0.01 - 100 |
| ENN A1/[^15^N_3_]-ENN A1 | 1.2175 – 0.0006 | 0.01 - 20 |
| ENN B/[^15^N_3_]-ENN A1 | 1.8991 + 0.0004 | 0.01 - 100 |
| ENN B1[^15^N_3_]-ENN A1 | 1.6541 –+0.0002 | 0.01 - 100 |
| Bea/[^15^N_3_]-ENN A1 | 1.8771 + 0.0001 | 0.01 - 100 |

1. **Spike Levels Validation**

|  | LOD and LOQ  [µg/kg] | | | | | Precision  [µg/kg] | Recovery  [µg/kg] | | | |  |
| --- | --- | --- | --- | --- | --- | --- | --- | --- | --- | --- | --- |
| Analyte | Level 1 | Level 2 | Level 3 | Level 4 |  | | Level 1 | Level 2 | Level 3 | Level 4 | |
| AOH | 0.30 | 0.70 | 1.00 | 3.00 | 5.00 | | 3.00 | 5.00 | 10.0 | 20.0 | |
| AOH-3-G | 0.10 | 0.30 | 0.70 | 1.00 | 5.00 | | 1.00 | 5.00 | 20.0 | / | |
| AOH-9-G | 0.20 | 0.60 | 1.40 | 2.00 | 5.00 | | 1.50 | 5.00 | 20.0 | / | |
| AOH-3-S | 0.02 | 0.06 | 0.14 | 0.20 | 5.00 | | 0.20 | 1.00 | 5.00 | 20.0 | |
| AME | 1 | 3 | 7 | 10 | 2.00 | | 0.10 | 2.00 | 10.0 | 20.0 | |
| AME-3-G | 1.00 | 3.00 | 7.00 | 10.0 | 10.0 | | 7.00 | 10.0 | 20.0 | / | |
| AME-3-S | 0.05 | 0.15 | 0.35 | 0.50 | 5.00 | | 0.50 | 1.00 | 5.00 | 20.0 | |
| ATX I | 3.00 | 9.00 | 20.0 | 30.0 | 20.0 | | 9.00 | 20.0 | 30.0 | / | |
| TeA | 0.30 | 0.90 | 2.10 | 3.00 | 10.0 | | 3.00 | 10.0 | 20.0 | 50.0 | |
| TEN | 0.15 | 0.35 | 0.50 | 1.50 | 5.00 | | 0.50 | 5.00 | 20.0 | / | |
| DON | 0.50 | 1.50 | 3.50 | 5.00 | 50.0 | | 5.00 | 50.0 | 300.0 | 500.0 | |
| DON-3-G | 3.00 | 9.00 | 21.00 | 30.0 | 30.0 | | 9.00 | 21.0 | 30.0 | 50.0 | |
| 3-AcDON | 0.50 | 1.50 | 3.50 | 5.00 | 10.0 | | 5.00 | 10.0 | 20.0 | 50.0 | |
| NIV | 5 | 15 | 35 | 50 | 35.0 | | 35.0 | 50.0 | 100 | / | |
| Fus X | 1 | 3 | 7 | 10 | 10.0 | | 10.0 | 25.0 | 50.0 | / | |
| ZEN | 0.10 | 0.30 | 0.70 | 1.00 | 10.0 | | 1.00 | 10.0 | 30.0 | / | |
| T2 | 0.05 | 0.15 | 0.35 | 0.50 | 5.00 | | 0.50 | 5.00 | 20.0 | 50.0 | |
| HT2 | 0.20 | 0.60 | 1.40 | 2.00 | 5.00 | | 2.00 | 5.00 | 20.0 | 50.0 | |
| ENN A | 0.005 | 0.015 | 0.035 | 0.05 | 1.00 | | 0.05 | 1.00 | 20.0 | 50.0 | |
| ENN A1 | 0.003 | 0.009 | 0.021 | 0.03 | 1.00 | | 0.03 | 1.00 | 20.0 | 50.0 | |
| ENN B | 0.005 | 0.015 | 0.035 | 0.05 | 1.00 | | 0.05 | 1.00 | 20.0 | 50.0 | |
| ENN B1 | 0.005 | 0.015 | 0.035 | 0.05 | 1.00 | | 0.05 | 1.00 | 20.0 | 50.0 | |
| Beau | 0.005 | 0.015 | 0.035 | 0.05 | 1.00 | | 0.05 | 1.00 | 20.0 | 50.0 | |

1. **Toxin Content in all samples**

See Excel file
